# Factors associated with treatment of women with osteoporosis or osteopenia from a national survey

**DOI:** 10.1186/1472-6874-12-1

**Published:** 2012-01-06

**Authors:** Eric S Meadows, Beth D Mitchell, Susan C Bolge, Joseph A Johnston, Nananda F Col

**Affiliations:** 1 Global Health Outcomes, Eli Lilly and Company, Indianapolis, IN, USA; 2 Centocor Ortho Biotech, Horsham, NJ, USA; 3 University of New England, Biddeford, ME, USA and Shared Decision Making Resources, Georgetown, ME, USA

## Abstract

**Background:**

Health outcomes could be improved if women at high risk for osteoporotic fracture were matched to effective treatment. This study determined the extent to which treatment for osteoporosis/osteopenia corresponded to the presence of specific risk factors for osteoporotic fracture.

**Methods:**

This retrospective analysis of the United States 2007 National Health and Wellness Survey included women age ≥ 40 years who reported having a diagnosis of osteoporosis (69% of 3276) or osteopenia (31% of 3276). Patients were stratified by whether they were or were not taking prescription treatment for osteoporosis/osteopenia. Using 34 patient characteristics as covariates, logistic regression was used to determine factors associated with treatment.

**Results:**

Current prescription treatment was reported by 1800 of 3276 (54.9%) women with osteoporosis/osteopenia. The following factors were associated with receiving prescription treatment: patient-reported diagnosis of osteoporosis (versus osteopenia); previous bone mineral density test; ≥ 2 fractures since age 50; older age; lower body mass index; better physical functioning; postmenopausal status; family history of osteoporosis; fewer comorbidities; prescription insurance coverage; higher total prescription count; higher ratio of prescription costs to monthly income; higher income; single status; previous visit to a rheumatologist or gynecologist; and 1 or 2 outpatient visits to healthcare provider (vs. none) in the prior 6 months. Glucocorticoid, tobacco, and daily alcohol use were risk factors for fracture that were not associated with treatment.

**Conclusions:**

There is a mismatch between those women who could benefit from treatment for osteoporosis and those who are actually treated. For example, self-reported use of glucocorticoids, tobacco, and alcohol were not associated with prescription treatment of osteoporosis. Other clinical and socioeconomic factors were associated with treatment (e.g. prescription drug coverage and higher income) or not (e.g. comorbid osteoarthritis and anxiety) and could be opportunities to improve care.

## Background

Osteoporosis is a systemic skeletal disorder characterized by low bone mass, structural deterioration of bone tissue, and an increased vulnerability to low-trauma fractures [1,2]. In the United States (US), an estimated 10 million people are affected by osteoporosis, and an additional 34 million are believed to have low bone mass, or osteopenia, placing them at increased risk for osteoporosis [3,4]. Low bone mass density (BMD) results in 1.5 million fractures annually, exacting a national cost of 14 billion dollars, and often, a profound personal cost [3,5]. These fractures are associated with chronic pain, increased dependence, reduced mobility, deformity, depression, loss of self-esteem, increased rates of hospitalization, and heavy personal socioeconomic burden [3,5,6].

Several pharmacologic agents approved by US Food and Drug Administration (FDA), including bisphosphonates, raloxifene, estrogen with or without progestin, and teriparatide, have shown efficacy in slowing or halting bone loss and reducing fracture risk [7,8]. Targeting effective treatments for osteoporosis to individuals at high risk for fracture would prevent more fractures and avoid unnecessary treatment of those at low risk for fracture.

Many medical specialty societies, academic institutions, professional consortiums, and private non-profit organizations have published guidelines for management of osteoporosis, in which treatment decisions are based primarily on BMD test results in combination with individual patient characteristics such as advanced age, family history, and tobacco use [9-13]. The World Health Organization (WHO) developed a country-specific fracture risk assessment tool (FRAX) for predicting a patient's 10-year probability of osteoporosis-related fracture based on age, gender, previous fracture history, BMD, low body mass index (BMI), use of oral glucocorticoid therapy, osteoporosis secondary to another condition, parental history of hip fracture, current smoking, and alcohol intake of 3 or more drinks per day [14-16]. FRAX plays a pivotal role in guiding recommendations for treatment by the National Osteoporosis Foundation. While postmenopausal women or men over 50 with a T-score of -2.5 or lower at the hip or spine or with a prior hip or spine fracture should definitely be treated, the 2008 Clinician's Guide uses the absolute fracture risk calculated from the US-adapted FRAX algorithm to help determine whether to treat [17]; thresholds for treatment are a ≥ 3% probability of osteoporosis-related hip fracture or a ≥ 20% risk of any osteoporosis-related fracture in the subsequent 10 years [18,19]. Despite advances in the diagnosis and treatment of osteoporosis, it remains underrecognized and undertreated in the US [20].

The primary objective of this study is to identify the patient-reported factors that correlate with receipt or nonreceipt of prescription treatment for osteoporosis or osteopenia in women who report having been diagnosed with the condition. A secondary objective is to compare the factors identified in this study to known clinical risk factors for osteoporotic fracture, using risk factors included in the FRAX algorithm.

## Methods

### Design

Data for this analysis were obtained from the 2007 US National Health and Wellness Survey (NHWS) [21], an annual, cross-sectional study of healthcare attitudes, behaviors, and outcomes. NHWS data are obtained from a web-based consumer panel, sampled to reflect the total US adult population. Participants recruited through internet advertising agreed to receive email invitations to participate in online surveys in exchange for sweepstakes entry and reward points redeemable for consumer products. Inclusion criteria for participation in the 2007 US NHWS were age ≥ 18 years, residence in the US, and ability to read and write English. The sample is drawn from the panel maintained by Lightspeed Research and invitations to participate in the NHWS are sent regardless of health status. The number of respondents has steadily increased each year, from 16,619 in 1998 to 63,012 in 2007. All 50 states and the District of Columbia and represented. Results are adjusted to reflect the total adult population by using known population incidences for key subgroups and weighting variables (gender, age, and race/ethnicity) using data from the previous year's Current Population Survey (Annual Demographics File) of the US Census Bureau. The NHWS study protocol and questionnaire were reviewed and approved by the Essex Investigational Review Board, Inc. (Lebanon, NJ, US). All participants provided informed consent prior to beginning the survey, and all identifying information was removed.

### Participants

The current analysis was limited to data from female respondents, age ≥ 40 years, who reported a physician diagnosis of osteoporosis or osteopenia.

### Measurements

#### Demographic and Socioeconomic Characteristics

Demographic measures in the NHWS included age, race/ethnicity, marital status, and number of children in household. Age was categorized as 40 to 54 (reference), 55 to 64, 65 to 74, and ≥ 75 years. Race/ethnicity was categorized as white (reference), African American, Hispanic, and other. Marital status was categorized as married/partnered, single, divorced/separated, or widowed. Number of children under the age of 18 in the household was assessed as a discrete continuous variable.

Socioeconomic characteristics included education, income, and employment status. Education was categorized as either having or not having graduated from college. Annual 2006 household income was categorized ($0-24,999; $25,000-49,999; $50,000-74,999; $75,000-99,999; $100,000-124,999; $125,000-149,999; and ≥ $150,000) and treated as an ordinal variable, with mean substitution of missing values due to responses of "decline to answer." To adjust for potentially different characteristics of those who declined to answer, a dummy variable for missing income was included in the final model. Working status was coded as employed or not employed.

#### General Clinical Characteristics

Mental and physical functioning were evaluated using the summary measures of the standard 12-item Medical Outcomes Study Short Form survey, US version 2.0 (SF-12v2), a patient-rated metric that includes questions regarding general health, bodily pain, mental health, vitality, social functioning, role limitations due to physical health problems, and role limitations due to emotional problems over the 4 weeks prior to completing the survey. Like the 36-item Short Form survey, the SF-12v2 provides physical and mental summary scores normalized for the US adult population with a mean of 50 and a standard deviation of ± 10, where higher scores represent better functioning [22].

The assignment of osteoporosis or osteopenia was made based on the subject self-reporting a physician diagnosis of osteoporosis or osteopenia, and the year of diagnosis. Subjects were also asked to self-report receipt of bone mineral density scan, current use of prescription medications to treat osteoporosis/osteopenia, and the number of bone fractures since age of 50. All respondents who reported taking a prescription medication to treat osteopenia or osteoporosis were asked to identify the medications they used. Choices were presented as the branded names and included "Actonel", "Boniva", "Evista", "Forteo", "Fosamax", "Fosamax Plus D", "Miacalcin", "hormonal treatment", and "Other" (asked to specify using free text). Reclast (zoledronic acid) was not included as a possible response because it was not approved to treat osteoporosis until 2007 after the survey was initiated. Family health history of osteoporosis included affected parents, grandparents, siblings, or other blood relative.

Clinical characteristics assessed as continuous variables included years since osteoporosis/osteopenia diagnosis, days exercised in the past month, BMI, and total number of prescription medications used for all conditions. Menopausal status, cigarette use, daily alcohol use, family history of osteoporosis, and disability preventing employment were handled as dichotomous (yes/no) variables. Other dichotomous variables included having a diagnosis of osteoporosis (as compared to osteopenia), having had a BMD test, and having undergone a hysterectomy with oopherectomy. For participants age 50 years and older, the number of fractures (any kind) occurring after age 50 was coded as 0 (reference), 1, or ≥ 2, and missing values were included as a dummy variable. Glucocorticoid use included betamethasone, cortisone, dexamethasone, fludrocortisone, hydrocortisone, methylprednisolone, prednisolone, prednisone, or triamcinolone, regardless of route of administration and condition being treated. Duration or past history of glucocorticoid use were not available from the survey.

#### Comorbidity

Both physical and psychiatric comorbidities were assessed. Overall physical comorbidity was assessed using an adaptation of the Charlson Comorbidity Index (CCI) [23], in which scores are assigned based on the presence or absence of 22 conditions. Conditions in the NHWS were matched to and assigned the point values of comparable conditions in the CCI. The presence or absence of back pain and osteoarthritis, which are not included as part of the CCI, were considered separately due to the potential relevance to osteoporosis diagnosis and/or sequelae. In order to account for both physical and mental comorbidity, we included data on the presence of anxiety and depression.

#### Healthcare Utilization and Costs

Healthcare utilization included inpatient and outpatient resource use in the previous 6 months. Hospitalization was considered as a dichotomous (yes/no) variable. Visits to primary care physicians, endocrinologists, gynecologists, nurse practitioners, rheumatologists, and orthopedic surgeons were assessed (yes/no) individually. Total number of provider visits (1-2, 3-6, and ≥ 7 visits, with none as the reference group) was treated as categorical variable.

Prescription coverage and use of generic medication and other cost-cutting strategies were considered as dichotomous (yes/no) variables. Cost-cutting strategies included taking less medication, cutting tablets in half, and taking fewer pills than prescribed. Out-of-pocket monthly expenditure for prescription medication was calculated as a proportion of monthly income and handled as a continuous variable.

### Data Analysis

#### Univariate Analyses

Descriptive statistics were calculated for all participants to determine the distribution of potential correlates. Patients were stratified by their use of prescription (versus no prescription) treatment for osteoporosis. All measures were compared between the prescription group and the no prescription group, using chi-square analyses for categorical variables and Student *t *tests for continuous variables. Tests of hypotheses were performed at a 2-sided significance level of 5%.

#### Multivariate Analyses

We conducted a logistic regression analysis to determine the independent association of factors that potentially correlate with receipt of prescription treatment for osteoporosis/osteopenia. The dependent variable was use of prescription treatment versus no prescription treatment. All covariates were included in the same regression model but are presented separately to facilitate discussion of 3 categories of possible predictors. Other than the fracture risk factors, other covariates included in the model were broadly classified as health status and behavior variables or demographic and socioeconomic characteristics. Missing data were handled as discussed in the description of the variables. Data analyses were conducted with SPSS^®^, version 12 (SPSS Inc., Chicago, IL).

## Results

**Figure 1:**
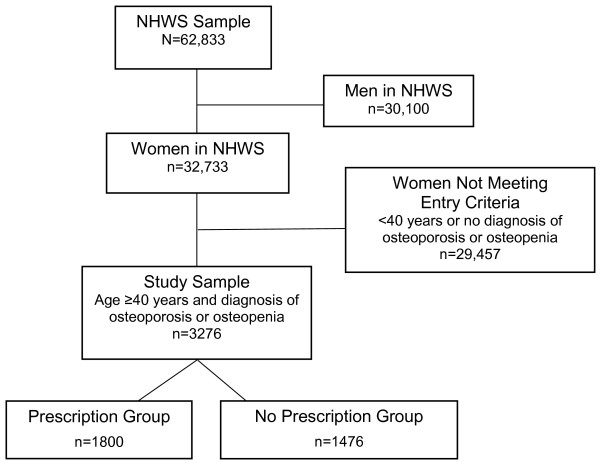
**Study population flow chart**. Abbreviations: n/N = number of patients; NHWS = National Health and Wellness Survey.

Of the 62,833 people included in the final NHWS dataset, 3276 (5.2%) were women over the age of 40 years who had been diagnosed with osteoporosis or osteopenia. Of these, 1800 (54.9%) reported using prescription medication for osteoporosis/osteopenia (Figure 1). The characteristics of the participants are summarized in Table 1. More women had osteoporosis (69%) than osteopenia (31%). About half reported having a family history of osteoporosis and approximately 30% had experienced a fracture themselves. Over 85% of the study sample reported having undergone BMD testing and most women described themselves as postmenopausal (69.2%). Nearly all participants (90.5%) were white and nearly a third had a college degree. Over 80% reported having prescription drug coverage.

**Table 1:** Characteristics of women with osteoporosis or osteopenia diagnosis according to prescription treatment status

	Study Sample (N = 3276)	No Prescription (n = 1476)	Prescription Treatment (n = 1800)	P value
**Risk factors for fracture**				

Age (mean)	64.4	63.3	65.2	< .001

Fracture history, %				

None	62.9	64.2	61.7	.14

1	15.3	14.7	15.8	.37

≥ 2	14.1	11.0	16.6	< .001

Osteoporosis, %	69.0	63.3	73.6	< .001

BMI (mean)	28.0	28.7	27.5	< .001

Family history of osteoporosis, %	50.6	49.1	51.9	.10

Glucocorticoid use, %	5.8	5.6	5.9	.64

Tobacco use, %	21.3	24.1	19.1	.001

Daily alcohol use, %	5.7	5.6	5.8	.73

**Health Status**				

SF-12v2 score				

Physical summary	40.4	39.1	41.4	< .001

Mental summary	49.1	48.3	49.7	< .001

Exercise in past month, d	6.8	6.4	7.2	.01

Years since diagnosis	6.4	6.3	6.5	.48

BMD testing, %	85.5	79.3	90.6	< .001

Postmenopausal, %	69.2	64.4	73.1	< .001

Hysterectomy with oophorectomy, %	29.1	28.6	29.6	.55

Comorbidity index score	1.02	1.07	0.97	.04

Prescription medications currently using for conditions other than osteoporosis, median	5.1	5.0	5.2	.13

Back pain, %	32.1	36.0	28.8	< .001

Osteoarthritis, %	50.5	55.6	46.3	< .001

Anxiety, %	25.2	30.3	21.1	< .001

Depression, %	24.0	26.6	21.9	.002

Disabled, %	8.0	8.7	7.4	0.18

Hospitalized (past 6 months), %	12.5	12.3	12.7	.69

Provider visits (6 months),%				

PCP	84.2	81.7	86.2	.001

Endocrinologist	5.3	4.9	5.6	.40

Gynecologist	16.0	14.3	17.4	.02

Nurse practitioner	15.0	15.4	14.7	.53

Orthopedist	12.8	13.1	12.6	.69

Rheumatologist	9.0	7.5	10.2	.008

Provider visits (6 months), %				

1-2	45.2	41.7	48.2	< .001

3-6	35.6	36.7	34.8	.27

≥ 7	10.9	10.8	10.9	.92

**Demographic and Socioeconomic Characteristics**				

Race/ethnicity, %				

White	90.5	89.8	91.0	.27

African American	3.5	3.0	3.9	.17

Hispanic	2.7	3.1	2.3	.17

Other	3.3	4.0	2.7	.04

Graduated college, %	27.7	25.7	29.4	.02

Employed, %	25.3	27.6	23.4	.005

Prescription coverage, %	82.9	79.0	86.1	< .001

Income (thousand dollars/y), %				

< 25	24.9	27.2	23.1	.02

25- < 50	30.7	31.4	30.1	

50- < 75	17.9	18.0	17.9	

75- < 100	8.2	7.1	9.1	

100- < 125	3.2	2.6	3.7	

125-150	1.7	1.8	1.6	

≥ 150	2.4	2.2	2.6	

Out-of-pocket spending on prescriptions as a proportion of income	0.05	0.05	0.06	.05

Cost cutting actions, %	16.4	17.8	15.3	.05

Ask for generic, %	36.9	36.2	37.4	.48

Children in home	0.18	0.22	0.15	.002

Marital status				

Married/partnered	56.9	57.7	56.2	.39

Single	4.3	4.1	4.6	.49

Divorced/separated	20.8	22.0	19.9	.14

Widowed	17.9	16.2	19.3	.02

Abbreviations: BMD = bone mineral density; BMI = body mass index; d = days; N = total number in study sample; n = number in subgroup; PCP = primary care provider; SF-12v2 = Short Form-12, version 2.0; y = year.

Participants who received prescription treatment for osteoporosis/osteopenia differed from those not receiving treatment as follows: older age, lower BMI, less tobacco use, better general health (e.g. SF-12 score), more days with exercise, fewer comorbidities, more highly educated, more commonly widowed, higher income, less likely to be employed, and better prescription drug coverage.

After adjusting for differences in measured characteristics between the treated and untreated groups, several factors were associated with receiving treatment. The odds ratios for receiving treatment are presented in Tables 2, 3, and 4. Some established risk factors were correlated with treatment: older age, lower BMI, having had 2 or more fractures after age 50, and a family history of osteoporosis (Table 2). Having a diagnosis of osteoporosis (vs. osteopenia) was also significantly correlated with treatment. Current glucocorticoid use, current tobacco use, and daily alcohol use were not correlated with treatment, even though these are established risk factors for fracture.

**Table 2:** Association between risk factors for fracture and prescription treatment of osteoporosis/osteopenia

		95% Confidence Interval		
	Odds Ratio	Low	High	P value
Age cohort (y)				
40-55	Reference			
55-64	1.33	0.98	1.79	.07
65-74	1.43	1.05	1.94	.02
≥ 75	1.17	0.79	1.72	.44

Fracture history				
0	Reference			
1	1.02	0.82	1.26	.86
≥ 2	1.50	1.19	1.88	.001

Osteoporosis (vs. osteopenia)	2.08	1.75	2.47	< .001

BMI	0.98	0.97	0.99	.003

Family history of osteoporosis	1.22	1.05	1.42	.01

Glucocorticoid use	0.84	0.60	1.18	.32

Tobacco use	0.84	0.69	1.02	.08

Daily alcohol use	0.87	0.63	1.20	.39

Abbreviations: BMD = bone mineral density; BMI = body mass index.

Variables listed in Tables 2, 3, and 4 were all included in the same regression model but are presented separately to highlight the 3 different groups of factors.

**Table 3:** Association between health status and behavior variables and prescription treatment of osteoporosis/osteopenia

		95% Confidence Interval		
	Odds Ratio	Low	High	P value
SF-12v2 score				
Physical summary	1.02	1.01	1.03	< .001
Mental summary	1.00	0.99	1.01	.76

Exercise in past month, d	1.00	0.99	1.01	.74

Years since diagnosis	1.00	0.98	1.01	.58

BMD testing	3.37	2.32	4.88	< .001

Postmenopausal	1.22	1.01	1.46	.04

Hysterectomy with oophorectomy	0.98	0.83	1.15	.78

Comorbidity index score	0.92	0.86	0.98	.02

Number of prescription medications currently using for conditions other than osteoporosis	1.07	1.04	1.09	< .001

Back pain	0.92	0.77	1.11	.40

Osteoarthritis	0.69	0.59	0.81	< .001

Anxiety	0.63	0.51	0.78	< .001

Depression	1.03	0.82	1.30	.77

Disabled	1.29	0.93	1.78	.13

Hospitalized (past 6 months)	1.10	0.86	1.39	.45

Provider visits by type				
PCP	1.05	0.77	1.41	.77
Endocrinologist	1.11	0.79	1.56	.55
Gynecologist	1.31	1.05	1.63	.02
Nurse practitioner	1.00	0.80	1.25	> .99
Orthopedist	1.00	0.78	1.28	> .99
Rheumatologist	1.40	1.05	1.86	.02

Provider visits by number				
0	reference			
1-2	1.69	1.13	2.52	.01
3-6	1.38	0.88	2.17	.16
≥ 7	1.41	0.82	2.42	.22

Abbreviations: d = days; PCP = primary care provider; SF-12v2 = Short Form-12, version 2.0.

Variables listed in Tables 2, 3, and 4 were all included in the same regression model but are presented separately to highlight the 3 different groups of factors.

**Table 4:** Association between demographic and socioeconomic variables and prescription treatment of osteoporosis/osteopenia

		95% Confidence Interval		
	Odds Ratio	Low	High	P value
Race/ethnicity				
White	Reference			
African American	1.34	0.88	2.05	.17
Hispanic	0.92	0.57	1.47	.73
Other	0.82	0.53	1.25	.35

Graduated college	1.09	0.92	1.31	.32

Employed	0.88	0.73	1.07	.21

Prescription coverage	1.49	1.22	1.82	< .001

Income ($25,000 unit)	1.08	1.01	1.15	.04

Out-of-pocket spending on prescriptions as a proportion of income	2.42	1.21	4.84	.01

Cost cutting actions	0.95	0.77	1.16	.60

Ask for generic	1.06	0.90	1.24	.48

Children in home	0.92	0.81	1.05	.21

Marital status				
Married	reference	reference	reference	
Single	1.49	1.02	2.20	.04
Divorced/separated	1.11	0.91	1.36	.31
Widowed	1.20	0.97	1.50	.10

Variables listed in Tables 2, 3, and 4 were all included in the same regression model but are presented separately to highlight the 3 different groups of factors.

We identified the following health status and behavior factors (Table 3) associated with prescription treatment: higher SF-12v2 Physical Summary score, having a BMD test in the past (regardless of result), postmenopausal status, lower comorbid burden, increased number of non-osteoporosis related prescription medications, absence of osteoarthritis, absence of anxiety, number of visits to gynecologists or rheumatologists in the past 6 months, and 1 to 2 (versus none) health care provider visits in the past 6 months. Socioeconomic and demographic variables (Table 4) that were significantly associated with receiving treatment included having prescription coverage, higher income, greater proportion of monthly income spent on prescription medication, and being single (vs. married).

## Discussion

Our analyses suggest that there is a substantial mismatch between those women who could benefit from treatment for osteoporosis and those who are actually treated. Women who are older, with a previous fracture, lower BMI, and family history of osteoporosis are being appropriately targeted for treatment. For example, these 3 risk factors are used to predict fracture risk in FRAX, an evidence-based risk model sponsored by the World Health Organization. Women with other established risk factors (glucocorticoid use, tobacco use, and daily alcohol use) were not more likely to be treated.

Patients with osteoarthritis and anxiety were less likely to receive prescription therapy for osteoporosis, regardless of their risk for osteoporotic fracture. It may be that chronic mental and physical pain conditions such as these compete and win for the clinician's attention over more silent conditions like osteopenia and early osteoporosis. Likewise, patients with chronic illness facing ongoing physical, emotional, and economic burden may prioritize treatment of a more painful condition such as osteoarthritis over a less painful one. With respect to osteoarthritis, the bone loss might appear to be less severe due to the underlying physical effects of osteoarthritis. The inverse association between osteoarthritis and osteoporosis might partially explain why patients with osteoporosis and osteoarthritis were less likely to receive treatment for osteoporosis [24,25], though it does not fully explain lower levels of treatment among those at high fracture risk. Anxious patients may be more reluctant to take medication because they may be more concerned about treatment side effects than non-anxious patients.

Our findings suggest that treatment patterns for osteoporosis depend on other socioeconomic issues such as income, having prescription drug coverage, and health care utilization. Although we weren't able to delineate these issues, we hypothesize that some factors, like comorbid osteoarthritis, might play an important role when the decision is made (or not) to initiate therapy. Others, like prescription drug coverage or income could be associated with persistence, or lack thereof.

In addition to providing an analysis of treatment patterns in relation to fracture risk, we built upon prior studies of prescription drug use in osteoporosis by examining a very large survey sample and including a wide breadth of patient-reported data [20]. In a meta-analysis addressing guideline adherence, predictors of treatment, and programs to assess improved care, Solomon et al. reported that no factor consistently predicted treatment [26]. Brennan et al. identified college education, higher income, more frequent medical care, and care by a gynecologist as correlates of prescription treatment [27]. Various other studies have shown that lower income is associated with lower BMD testing and prescription treatment in managed care settings [28], among patients with Medicare [29], and within a national healthcare system [30]. In another study of glucocorticoid-induced osteoporosis, patients with more comorbid conditions were generally less likely to receive prescription treatment for osteoporosis than patients with less comorbid burden. Only 42% of patients who were taking glucocorticoids received prescription treatment, and only 23% underwent bone densitometry [31].

Our study has a number of important limitations. One was the absence of BMD test results or clinical information from medical records, such as were analyzed in a recent study that provides complimentary information to our work [32]. The self-report of medical procedures, like a BMD test, could be prone to a lack of subject understanding and recall bias. Additionally, the use of glucocorticoids was only qualitatively addressed and did not include former use. A substantial proportion of patients might be using low doses, inhaled formulations, or short durations of therapy that might not significantly affect their fracture risk; others might have previously used glucocorticoids for extended periods of time. Our risk factor variables were not sufficient to reliably calculate each patient's risk of fracture. One particularly important difference is that our study included all routes of administration while predictive models like FRAX include only oral glucocorticoids. However, previous studies have demonstrated the undertreatment of patients using glucocorticoids [31,33], which is consistent with our findings. We could not determine at the individual patient level which patients who needed treatment received it and which patients were being treated unnecessarily because we were unable to examine the composite risk for fracture of individual patients. Our sample provides no information on patients with undiagnosed osteoporosis or osteopenia, who are estimated to comprise over 50% of those with the condition [34], The small numbers of non-white participants limited our ability to detect differences between groups based on race or ethnicity. Questions on the NHWS did not allow us to distinguish between undertreatment, problems with initiating therapy, or lack of persistence, which has been shown to be a significant problem in treatment of this disease [35], Finally, the cross-sectional study design used in this analysis did not permit inferences regarding causality.

## Conclusions

Efforts to reduce the association between undertreatment and lower income and lack of prescription coverage are warranted, especially as treatments become less expensive with the introduction of generic bisphosphonates in the US. Further research is needed to understand how fracture risk correlates with evidence-based clinical decisions. Some risk factors correlated with treatment while others did not and thus it would be valuable to identify and address the barriers to treatment that exist for women who smoke or drink heavily.

## Competing interests

ESM, BDM, and JAJ are employees of Eli Lilly and Company and hold stock and/or stock options. SCB is currently employed by Centocor Ortho Biotech and was employed by Consumer Health Sciences at the time of this study. NFC has received honoraria from the Advisory Council on Evidence, Quality, and Value, Boehringer Ingelheim Pharmaceuticals, Inc. The National Health and Wellness Survey (NHWS) is conducted by Consumer Health Sciences, Princeton, NJ. Eli Lilly and Company, Indianapolis, IN licensed access to NHWS. Financial support was provided by Eli Lilly and Company, Indianapolis, IN, USA. Eli Lilly and Company markets two treatments for osteoporosis, raloxifene (Evista) and teriparatide (Forteo).

## Authors' contributions

ESM, JAJ, and SCB made substantial contributions to conception, design, analysis and interpretation of data. BDM and NC made substantial contributions to the design of the study and interpretation of the data. SCB drafted the first version of the manuscript. ESM made the first major revision and led the subsequent revision process. All authors were involved in revising the drafts critically for important intellectual content and gave final approval of the version to be published.

## Pre-publication history

The pre-publication history for this paper can be accessed here:

http://www.biomedcentral.com/1472-6874/12/1/prepub
